# Patient involvement in assessing consultation quality: validation of patient enablement instrument (PEI) in Lithuanian general practice

**DOI:** 10.1186/s12875-019-1061-1

**Published:** 2019-12-03

**Authors:** Aelita Skarbalienė, Arnoldas Jurgutis, Eva Lena Strandberg, Teresa Pawlikowska

**Affiliations:** 1 Faculty of Health Sciences, Klaipeda University, H.Manto, 84 Klaipeda, Lithuania; 2 Faculty of Medicine, Lund University, Jan Waldenströms gata, 35 Malmö, Sweden; 3 Health Professions Education Centre, RCSI, 123 St. Stephen’s Green, Dublin 2, Ireland

**Keywords:** Patient enablement, Patient enablement instrument (PEI), General practice, Primary health care, Patient centeredness, Consultation, Quality

## Abstract

**Background:**

The Patient Enablement Instrument (PEI) was designed to encapsulate consultation outcome from the perspective that increasing their understanding and coping ability would underpin a positive consultation outcome for patients. The objective of the study was the validation of the PEI in Lithuanian general practice and comparison of Lithuanian patients’ enablement with previous studies in Europe to see if factors associated with patient enablement in Lithuania were reflective of those in the previous studies.

**Methods:**

The Patient Enablement Instrument was translated into Lithuanian and included in the questionnaire along with the questions about a person’s health, reasons for visiting the doctor and feeling about the consultation. Practices from 4 different municipalities that are situated in different geographical regions which have both town and rural areas were sampled randomly. Patients scheduled consecutively aged 18 years or more were the subjects of the study. The data analyses focused on internal reliability and concept validity.

**Results:**

The overall mean patient enablement score was 6.43. Enablement scores declined with increasing patient age, and female patients were more enabled. Patients with biomedical problems had the highest enablement results, while patients with complex problems had the lower results. Enablement was positively related to receiving a prescription and knowing a doctor, and negatively related to wish having consultation with another doctor.

**Conclusions:**

This study substantiates the rationality of using PEI in assessing primary care consultations in Lithuania. The correlations of enablement largely reflect the situation in Western and Central Europe: longer consultation and access to the same physician increases patient enablement.

## Background

Patient-doctor consultations are pivotal to the delivery of high quality patient care, nowhere more so than in the first contact care that is family practice characterized by people presenting with undifferentiated problems. The essence of primary care has been characterized as being holistic and patient – centered, and measures have been developed to capture this: one of the earliest, patient satisfaction, although popular can be seen as an amalgamation of a number of facets of health care experienced by patients, and so the Patient Enablement Instrument (PEI) was derived from reviewing the literature and working with patients to capture a more directly patient centered focus for consultation quality. Patient enablement can be defined as the extent to which a patient is capable of understanding and coping with his or her health issues. This concept is linked to a number of health outcomes such as self-management of chronic diseases and quality of life [1], it is an indicator of the self-efficacy benefits of consulting a health care provider and is expected to be associated with behaviours like treatment adherence and self-care and indicators of quality of care [2]. PEI was designed to encapsulate consultation outcome from the perspective that increasing their understanding and coping ability would underpin a positive consultation outcome for patients [3]. Howie et al. also demonstrated that the PEI differed from, but was related to patient satisfaction measures [3].

The PEI has now been translated and validated in a number of countries with broadly similar results. Patients’ enablement scores increases in value with patient-doctor continuity of care [3–6], consultation duration [3, 4, 6, 7], and receiving a prescription when one is expected [4, 7]. Doctors’ communication skills and perceived empathy have also been shown to relate to better patient enablement [4]. Age, health needs and culture have also been shown to relate to PEI outcome, although there is some inconsistency [3, 4, 6].

The aim of this study was the validation of the PEI in Lithuanian general practice and comparison of Lithuanian patients’ enablement with previous studies in Europe to see if factors associated with patient enablement in Lithuania were reflective of those in the previous studies.

## Methods

### Setting

This was the first cross-sectional study using the Patient Enablement Instrument (PEI) in Lithuanian primary care. After joining EU in 2004, Lithuania committed to harmonize regulation of its primary health service. Lithuanian general practitioners (GPs) were relatively doctor-centered, then with harmonization and accession to EU one aim was to increase patient-centeredness [8]. The Scandinavian model was chosen for implementation where consultation length is about 20 min and several health problems during one meeting can be discussed.

### Recruitment of general practices

Four different municipalities that are situated in different geographical regions, which have both town and rural areas, were sampled randomly and every single practice was invited to participate in the research. Sample size was estimated from prior works [3, 9] to ensure that we captured the expected diversity 50 consultations were sampled per doctor and 50 doctors participated in the research.

### Recruitment of patients

Patients scheduled consecutively aged 18 years or more were asked to participate in the research. The receptionist asked each respondent to fill-in the questionnaire when he / she came to see the doctor. Receptionists at the primary health centers were trained how to ask people to fill-in the questionnaires and gain informed consent: how to explain the objectives of the research and how to emphasise the principles of voluntariness and anonymity.

### The instrument

Patient Enablement Instrument (PEI) is a six-item, three-scale questionnaire about patients’ perceptions of their ability to understand, cope, and manage with their illnesses after a consultation [1]. The items in the questionnaire ask the degree to which patients - after they have had a consultation with a GP - feel able to: 1) understand their problem(s)/illness; 2) cope with the problem(s)/illness; 3) keep themselves healthy; 4) cope with life; 5) be confident about their health; and 6) help themselves. The scale in the PEI is “much better/more” (2 points), “better/more” (1 point), “same or less” (0 point), and “not applicable” (0 points), leading to a sum score ranging from 0 to 12. This PEI score could be calculated when at least three of six questions had been answered. There is not a clear consensus which PEI score is considered “good” or “adequate”. The PEI was translated into Lithuanian as part of the questionnaire. The PEI was translated from English to Lithuanian, then was back-translated by another translator and proofread by an English native speaker. After an initial reliability (pilot) study, it was decided to use the PEI unchanged for the main study. Only one word in the PEI questionnaire was changed. “Cope” has few synonyms in Lithuanian language, but the main option is *susidoroti*. Linguistically it is not used when collocated with ‘illness’. The discussion with the group of doctors was held after the pilot study in order to get their opinions and the consensus was to use synonym of “cope” - *įveikti*, because it is more usual in context.

Beside the six PEI questions, the questionnaire asks demographic details, the reasons for visiting the doctor, expectations about the consultation (expectations to get the prescription), and continuity of care (knowing the doctor and preference to see another doctor). The reasons for visiting the doctor were formulated according the ‘needs’ categories defined by Howie et al. [3], e.g. biomedical, social, psychological, administrative (getting a medical history statement, a confirmation about the health status, etc.) or complex of previously mentioned needs.

The questionnaire was anonymised for patients. In order to comply with the requirements for research ethics and get the trust of the respondents every questionnaire was packed in a separate envelope.

Questions about person’s health and reasons for visiting the doctor were answered while waiting for the consultation. Doctors marked-in the date of the consultation, as well as consultation time in and time out. Immediately after the consultation, respondents answered the PEI questions.

Only questionnaires that were completely filled-in were used for the analysis.

### Data analysis

The data from the questionnaires were entered into SPSS version 20 database manually. The data analysis was carried out using the SPSS software version 20. Cronbach’s alphas were calculated to determine internal consistency between questionnaire items, factor analysis was used to find the internal construct validity, and the Mann-Whitney U test was used to compare the equivalence of distributions.

## Results

A total of 2500 consulting patients participated with 94% (2342) of full completion of the questionnaire indicating good acceptability. Another 23 were not completely filled-in and were not included into the analysis. The patients’ characteristics can be seen in Table 1.

**Table 1:** The patients’ characteristics

Indicator	Total (*n* = 2342)
Age, mean (SD)	53.43 (15.08)
Gender, N (%)	
Men	808 (34.5)
Women	1534 (65.6)
Need for visiting a doctor, N (%)	
Biomedical	1342 (57.30)
Social	62 (2.65)
Psychological	98 (4.18)
Complex	644 (27.50)
Administrative	196 (8.37)
Medicine prescription, N (%)	
Expecting the prescription	1761 (75.2)
Getting the prescription	1586 (67.7)
Knowing the doctor, N (%)	
Very well	1272 (54.3)
Well	452 (19.3)
Average	302 (12.9)
A little bit	166 (7.1)
Not knowing	150 (6.4)
Visiting regular doctor, N (%)	2030 (86.7)
Prefer to see another doctor, N (%)	494 (21.1)

The value for Cronbach’s alpha in Lithuania was high (0.842) denoting a strong consistency between the PEI variables.

Results show moderate to strong correlations between each PEI item (from 0.451 to 0.801) and strong correlations between each item and the total score (from 0.741 to 0.921) (Table 2).

**Table 2:** Spearman correlation coefficients between PEI items and total score

Items	PEI	p
Able to cope with life	0.741	0.000
Able to understand your illness	0.921	0.001
Able to cope with your illness	0.839	0.000
Able to keep yourself healthy	0.811	0.000
Confident about your health	0.879	0.002
Able to help yourself	0.757	0.000

The overall mean patient enablement score was 6.43 (SD 4.1, PEI scores range 0–12). 152 respondents scored the floor points (0) and 943 study participants scored ceiling points (12).

Enablement differed when comparing age groups and gender (Table 3). Increasing age was associated with decreasing patient enablement scores (*p* < 0.05), and female patients were more enabled than the male patients were (*p* < 0.01).

**Table 3:** Mean enablement scores and demographics

Age groups	Mean PEI score (SD)
18–40 years	6.4 (3.4)
41–60 years	5.4 (2.9)
60 years and older	4.4 (2.4)
Gender	
Male	5.6 (3.2)
Female	6.9 (4.0)

Table 4 shows the mean enablement scores and duration of consultation for each of the patient self-reported needs. Enablement scores were the highest for biomedical problems and lower for complex problems (biomedical, psychological and social in combination). Enablement was the lowest for patients with administrative needs.

**Table 4:** Mean enablement score and duration of consultation by ‘needs’ category

Focus of self-reported consultation ‘need’	n (PEI)	Mean PEI score (SD)	Mean (SD) duration (min)
Biomedical	1342	6.9	16.8
Social	62	6.3	16.9
Psychological	98	6.6	17.7
Complex	644	5.7	15.2
Administrative	196	4.5	9.8
Total	2342	6.43 (4.1)	15.8 (4.3)

'needs' categories are as defined by Howie et al [3].

The mean of consultation duration was 15.8 min (SD 4.3) and mean duration of consultations in Lithuania were the highest in consultations that included a psychological component.

Enablement was also correlated with getting the prescription for medications (mean PEI when prescription was received 4.8 (SD 3.1); mean PEI when prescription was not received 3.1 (SD 3.0); Mann-Whitney U-test for equivalence of distributions standed *p* < 0.001).

Patient enablement had a positive linear correlation with knowing the doctor well, Spearman correlation coefficient of PEI with knowing the doctor was 0.38; *p* < 0.001. The mean of enablement for patients who preferred visiting a different doctor was 3.3 (SD 3.1); and the mean of enablement for patients who did not preferred a different doctor was 6.1 (SD 3.4); Mann–Whitney U-test for equivalence of distributions standed *p* < 0.001).

Patient enablement had also a positive correlation with the length of the consultation, Spearman correlation coefficient of PEI with length of the consultations was 0.54; *p* < 0.001). Thus knowing the doctor well and having longer consultation increased patient enablement.

As the PEI was based on theoretical model, the concept validity was verified using factorial analysis. The six PEI questions were introduced as target items. Principal axis factoring was selected as the method of extraction. Because we expected the two factors to be correlated, we selected Oblimin with Kaiser normalization for factor rotation. Eigenvalue and % of variance for the first factor are respectively 2.42, 35.4; and for the second factor 1.18, 17.13. Results indicate that the two factors extracted explain 52.53% of the total variance. The KMO index indicated that the factorial loading in two factors was statistically satisfactory (.79). This value indicates that the correlation patterns make it possible to clearly distinguish between the two factors. Table 5 shows the factorial loading between the items. It can be noticed that the last two questions separate the factors. And the correlation between the two factors is .48.

**Table 5:** Factor loadings of the items of the PEI

	Factor 1	Factor 2
Able to cope with life	0.77	0.63
Able to understand your illness	0.63	0.46
Able to cope with your illness	0.84	0.49
Able to keep yourself healthy	0.76	0.36
Confident about your health	0.54	0.80
Able to help yourself	0.57	0.74

## Discussion

Though the *enablement* term is usual in contemporary Lithuanian health care, this was the first report on the validity of the patient enablement instrument (PEI) in Lithuanian general practice.

In line with international experience, the results demonstrated good response rate of PEI by both patients and primary health centers with a response rate of 95%. The data from 94% of the questionnaires could be used for analysis and this proved that all patients, including the older ones, could understand and respond to the questions in few minutes: so the PEI is both acceptable and feasible in Lithuanian general practice.

The use of such patient completed instruments brings with it the uncertainty of reported outcome reflecting the patients’ internalized experience. The value for Cronbach’s alpha in Lithuania was high (> 0.8) and similar to other studies [4, 5, 7] supporting high internal consistency.

The average patient enablement reported was 6.43 and was very similar to the study in Croatia (mean score 6.6) [6] and higher than a similar study in Sweden (mean score 3.48) [5], Poland (mean score 4.0) [4], the UK (mean score 3.1) [3], Scotland (mean score 3.0) [7], and France (mean score 5.06) [1]. Since the sample sizes in the studies mentioned above were very similar, the differences in the level of patient enablement may be explained by cultural and linguistic differences, as Howie et al. discuss that social and cultural issues shape different patient expectations and influence enablement [3].

Regardless of the differences of enablement level, a statistical difference when comparing age groups and gender was revealed in most of the countries where PEI was validated. In Lithuania PEI scores declined with increasing patient age and female patients were more enabled than the male patients. Older age in male patients predicted low enablement in Croatia [6] and patient enablement was positively associated with female patients and younger age in Poland [4]. However, other study revealed that middle-aged patients (31–60 years) were significantly less enabled than the group aged 16–30 years. However, the negative association with increasing age did not hold among older patients (≥61 years) [10]. It was argued that enablement results with regard to the patient’s age are contradictory [11]. This allows questioning the influence of age on enablement.

Patient needs exhibited that patients consult family doctors mostly for biomedical or complex problems and that was similar to some findings in Western European countries. The enablement of patients, who consulted for biomedical problems was higher in Lithuania, and enablement decreased when the consultation was for complex or administrative problem. Similar tendencies were revealed in the UK study [3] and the Polish study reported that enablement scores were the highest for biomedical problems and lower for complex problems (biomedical, psychological and social in combination). Enablement was the lowest for patients with administrative needs [4]. Enablement was also independently negatively associated with the complexity of consultation (patients wishing to discuss psychological or social problems plus or minus physical problems) in Scotland [7].

Studies found a high rate of prescribing in Lithuania [12] and revealed that patient enablement increased when patients’ expectations of receiving a prescription were fulfilled, as in Poland and the UK studies [4, 7].

This study underlined the importance of knowing the doctor for patient enablement in Lithuania: this tendency supporting continuity of practitioners very similar to the experience of other countries. At individual consultations in the UK, knowing the doctor well was most closely associated with enablement score [3]. The lack of continuity of care predicted low enablement in Croatia [6]. The patient’s perception of continuity and the doctor’s communication skills were related to the higher PEI scores in Sweden [5], and knowing the doctor was independently related to patient enablement in the Polish context [4]. Mercer et al. however found that consultation length and continuity of care (knowing the doctor well) were not related to enablement in their last study in Scotland, but their previous work did find a weak positive association between enablement and continuity of care [7].

Consultation duration was another factor associated with the higher enablement in Lithuania: patient enablement rose with consultation duration. Sufficient consultation time is a global concern [3]. The longer mean consultation time associates with the increased enablement around the Europe. Enablement score was closely associated with duration of consultation in the UK [3], Croatia [6], Poland [4], and Scotland [7]. Worth noting that the value of continuity and longer duration of consultation was not only established for patients attending a GP in primary care, but also for those consulting with general practice nurses [13]. Thus, once again, this study underlined the importance of consultation duration for patient enablement in Lithuania, as in other countries.

### Limitations of the study

Test - retest reliability was not tested. Having in mind that one of some serious test - retest limitation is that the first test-taking experience may affect performance on the second test administration (individual may perform better at the second testing after having learned from the first experience). PEI is only six questions and respondents could learn them, so we believe we would not have obtained the reliable results if test - retest was performed. However, test - retest analysis could bring valuable results about the dilution of patient enablement over the time. Thus, the further research on patient enablement in Lithuania is needed.

The standard error of measurement was not tested either. Since standard error of measurement serves in a complementary role to the reliability coefficient, we rely only on the reliability testing in this study.

## Conclusion

This study justifies the use of the patient enablement instrument PEI for measuring the routine consultation quality in primary care in Lithuania: validity was good. The results of the PEI and its relationships with respondents’ characteristics replicate those found in Western and Central Europe, i.e. a longer consultation time and greater continuity of care increases patient enablement. Based on the results it is possible to make suggestions for improvement of GP practice, i.e. seeking continuity of care and longer consultations in primary care.

## Data Availability

The datasets used and/or analysed during the current study are available from the corresponding author on reasonable request.

## Abbreviations

GP: General practitioner
PEI: Patient Enablement Instrument
